# Dietary factors and sociodemographic determinants of non-communicable diseases among adults: evidence from a cross-sectional study

**DOI:** 10.3389/fpubh.2025.1688260

**Published:** 2025-10-21

**Authors:** Ali Mohieldin

**Affiliations:** Public Health Department, College of Science, King Khalid University, Abha, Saudi Arabia

**Keywords:** non-communicable diseases, dietary predictors, obesity, lifestyle, public health

## Abstract

**Background:**

Non-communicable diseases (NCDS) account for over 70% of global mortality. Integrated data on lifestyle and dietary risk factors remain limited in the middle east.

**Objective:**

To assess associations between sociodemographic, anthropometric, and dietary predictors and self-reported NCD status among adults in Saudi Arabia.

**Methods:**

A cross-sectional survey of 430 adults in Asir region province (July 2025) collected data on demographics, BMII, physical activity, and dietary intake across 10 food groups. NCDs were defined as physician-diagnosed cardiovascular disease, diabetes mellitus type 2, cancer, or chronic respiratory illness. Bivariate associations were evaluated using pearson’s *χ*^2^ tests; multivariable logistic regression identified independent predictors. IBM SPSS v29 was used.

**Results:**

NCD prevalence was 49.3%. Bivariate analysis showed age (*p* < 0.001), female gender (*p* = 0.045), marital status (*p* = 0.034), obesity (*p* < 0.001), and occupation (*p* = 0.004) were significant predictors. Low fruit (*p* = 0.033), dairy (*p* = 0.002), and grain intake (*p* = 0.014), and high sugary food intake (*p* = 0.009) were significantly associated with NCDs. Logistic regression indicated that female gender (OR = 2.87, 95% CI: 1.02–8.08), low dairy intake (OR = 0.21, 95% CI: 0.08–0.57), high sugar intake (OR = 0.10, 95% CI: 0.03–0.33), and smoking (OR = 0.35, 95% CI: 0.13–0.93) were significant independent predictors. Some findings were counterintuitive, warranting cautious interpretation.

**Conclusion:**

Nearly half of adults had at least one NCD. Modifiable dietary factors, notably fruit, dairy, grain, and sugar intake, emerged as key risk factors. Tailored dietary interventions are crucial.

## 1 Introduction

Non-communicable diseases (NCDs) are the leading cause of death globally, responsible for over 70% of total mortality, with cardiovascular diseases, diabetes, cancer, and chronic respiratory diseases accounting for most of these deaths (1). In Saudi Arabia, national reports indicate that nearly three-quarters of deaths are attributable to NCDs, reflecting the combined impact of rapid urbanization, sedentary lifestyles, and dietary transitions (2).

Dietary behaviors in the Middle East are undergoing profound changes, with high consumption of refined carbohydrates, fried foods, and sugar-sweetened beverages, alongside inadequate intake of fruits, vegetables, and dairy products (3, 4). Several Saudi-based studies highlight similar dietary challenges, including frequent fast-food consumption, low fruit and vegetable intake, and rising obesity rates (5–7). For example, Aljefree and Ahmed (3) systematically reviewed dietary factors in the Middle East and found that “Westernized” diets rich in sugar and fats were strongly linked with cardiovascular diseases. Similarly, Al-Nozha et al. and Alqarni reported alarming obesity levels in Saudi Arabia, closely tied to shifts in diet and physical inactivity (5, 6).

Globally, evidence supports the association between dietary intake and NCD risk. High consumption of fruits, vegetables, and dairy products has been shown to reduce the risk of cardiovascular and metabolic disorders (8, 9), while frequent intake of sugary foods and beverages is strongly linked with obesity, insulin resistance, and diabetes (10, 11). Despite this, studies integrating dietary intake behaviors with sociodemographic determinants remain scarce in Saudi Arabia and the wider Middle East. This gap underscores the importance of locally relevant research to inform culturally tailored interventions aligned with Saudi Vision 2030.

Globally, evidence supports the association between dietary intake and NCD risk. High consumption of fruits, vegetables, and dairy products has been shown to reduce the risk of cardiovascular and metabolic disorders (8, 9). For example, Aune et al. (8) demonstrated that fruit and vegetable intake is inversely associated with cardiovascular disease, cancer, and all-cause mortality, while Gijsbers et al. (9) reported that dairy consumption, particularly low-fat dairy, was linked with reduced risk of type 2 diabetes. Conversely, frequent intake of sugary foods and beverages is strongly linked with obesity, insulin resistance, and diabetes (10, 11). Supporting this, it was found that sugar-sweetened beverage intake significantly increases type 2 diabetes incidence (10), also it was confirmed that excess dietary sugars contribute to weight gain and obesity in both randomized controlled trials and cohort studies (11)> Collectively, these findings highlight the importance of monitoring dietary intake frequencies and their contribution to chronic disease risk. Despite this robust global evidence, studies integrating dietary behaviors with sociodemographic determinants remain scarce in the Middle East and Saudi Arabia, underscoring the urgent need for locally relevant research to inform culturally tailored interventions aligned with Saudi Vision 2030.

## 2 Methods

### 2.1 Study design and participants

Participants (*n* = 430) were recruited via convenience sampling in Asir region province, Saudi Arabia, in July 2025. Inclusion criteria were adults aged ≥18 years who provided informed consent.

#### 2.1.1 Variables

Outcome: presence of ≥1 self-reported physician-diagnosed NCD (CVDS, diabetes, High blood pressures, cancer, GI disorder, and Osteoporosis).

#### 2.1.2 Dietary assessment tool

Dietary intake frequencies were assessed using a structured questionnaire covering 10 food groups, adapted from the Food and Agriculture Organization (FAO) dietary diversity guidelines (12) and previously applied in nutrition surveillance studies in the Middle East (3, 7). Intake frequencies were categorized into adequate and inadequate consumption based on WHO dietary recommendations (13, 14).

### 2.2 Questionnaire validation

The questionnaire was based on internationally validated FAO/WHO dietary diversity instruments (12, 13). In the current study, a pilot test with 25 adults from the same region was conducted to assess clarity, cultural appropriateness, and timing, with minor modifications made based on feedback. Internal consistency of the dietary intake items was acceptable (Cronbach’s alpha = 0.82). Content validity was supported by prior use of similar instruments in Saudi and Middle Eastern populations (3, 4, 7). Although, formal linguistic validation was not performed, the questionnaire was provided in Arabic and English versions reviewed by bilingual public health experts to ensure clarity.

Predictors: age, gender, marital status, BMII category (WHO), education, occupation, smoking status, residence type, income, physical activity, Dietary factor (10 food groups × 5 frequency levels), and meal frequency.

### 2.3 Sample size calculation

The minimum required sample size was estimated using Cochran’s formula for prevalence studies:

$n=\frac{Z^{2}\cdot p(1-p)}{d^{2}}$

Assuming a 50% prevalence of NCDs (to maximize sample size), a 95% confidence interval (*Z* = 1.96), and a margin of error of 5% (*d* = 0.05), the required sample was 384. To account for an anticipated 10–12% non-response rate, the final target was increased to ≥420, with 430 adults included in this study.

Participants were recruited using a convenience sampling approach. This method was selected due to practical and logistical considerations, including limited time and resources, as well as the absence of a comprehensive population registry in the region to support probability-based sampling. Convenience sampling also allowed rapid recruitment across different age groups, occupations, and residential settings, which was important for achieving the target sample size within the study timeline. To mitigate potential bias inherent in convenience sampling, trained public health students conducted face-to-face interviews with participants to ensure accurate comprehension of questions and minimize response errors. While this approach may still introduce selection bias and limit generalizability, it was deemed appropriate for exploratory cross-sectional research aimed at generating preliminary evidence to inform larger, probability-based studies in the future.

### 2.4 Inclusion and exclusion criteria

Eligible participants were Saudi adults (≥18 years) residing in the Asir region who provided informed consent. Adults with existing family history of NCDs were not excluded, as the aim was to capture population-level prevalence and determinants, and family history is an important background factor. Individuals with minor physical limitations were included provided they could complete the questionnaire. However, adults with severe cognitive or physical disabilities that precluded independent completion of the questionnaire, as well as those with diagnosed eating disorders, were excluded to avoid response bias and misclassification. This approach ensured inclusivity while preserving data quality.

### 2.5 Anthropometric measures

Participants self-reported their weight and height, which were used to calculate body mass index (BMI) as weight (kg) divided by height in meters squared (kg/m^2^). BMI categories followed the World Health Organization (WHO) cutoffs: normal (<25 kg/m^2^), overweight (25.0–29.9 kg/m^2^), and obese (≥30 kg/m^2^) (15).

### 2.6 Dietary assessment tools and scoring

Dietary intake frequencies were assessed using a structured questionnaire covering 10 food groups (fruits, vegetables, grains, dairy products, legumes, meat, fish, oils, sugary foods, and beverages). The tool was adapted from the Food and Agriculture Organization (FAO) guidelines for measuring household and individual dietary diversity (12). Frequency of intake was recorded on a five-point scale (daily, 3–5 times/week, 1–2 times/week, rarely, never). For analytic purposes, consumption of protective foods (e.g., fruits, vegetables, dairy) was classified as *adequate* if intake was ≥3 times per week, and *inadequate* otherwise, consistent with FAO and WHO recommendations (12, 15). Conversely, frequent consumption of discretionary items (sugary foods, fast food) was categorized as *unhealthy* if ≥3 times per week.

### 2.7 Statistical analysis

Bivariate associations were examined via pearson’s χ^2^ tests. Cramér’s v was used to estimate effect sizes. Logistic regression using the enter method assessed independent predictors. Statistical significance was set at *α* = 0.05. IBM SPSS v29 was used.

## 3 Results

### 3.1 Results summary

The study sample consisted of 430 adult participants from the Asir region of Saudi Arabia. As shown in Table 1, females comprised 59.5% of the sample and males 40.5%. The majority of participants were between 25 and 44 years of age (57.2%), followed by those aged 45 years and above (24.7%), and those aged 24 years or younger (18.1%). Regarding body mass index (BMI), 47.9% of participants had a normal BMI (<25), 27.0% were overweight (25–29.9), and 25.1% were obese (≥30). More than half of participants were married (55.8%), while 44.2% were single.

**Table 1:** Sociodemographic and lifestyle characteristics of participants (*n* = 430).

Variable	Categories	*n* (%)
Age group (years)	18–25	45 (10.5)
	26–35	130 (30.2)
	36–45	164 (38.1)
	>45	91 (21.2)
Gender	Male	174 (40.5)
	Female	256 (59.5)
Marital status	Single	134 (31.2)
	Married	296 (68.8)
BMI category	Underweight (<18.5)	5 (1.2)
	Normal (18.5–24.9)	110 (25.6)
	Overweight (25–29.9)	207 (48.1)
	Obese (≥30)	108 (25.1)
Education level	≤Secondary	68 (15.8)
	Graduate	317 (73.7)
	Postgraduate	45 (10.5)
Occupation	Unemployed	104 (24.2)
	Government employee	227 (52.8)
	Private sector	62 (14.4)
	Student	24 (5.6)
	Retired	13 (3.0)
Residence	Urban	362 (84.2)
	Rural	68 (15.8)
Household income	<5,000 SAR	49 (11.4)
	5,000–10,000 SAR	237 (55.1)
	>10,000 SAR	144 (33.5)
Smoking	Yes	61 (14.2)
	No	369 (85.8)
Physical activity	None	155 (36.0)
	Limited	228 (53.0)
	Regular	28 (6.5)
	Heavy/exceptional	19 (4.4)

Values are presented as frequency (*n*) and percentage (%). Percentages are calculated based on the total sample (*n* = 430). BMI categories follow WHO cutoffs: underweight (<18.5 kg/m^2^), normal (18.5–24.9 kg/m^2^), overweight (25.0–29.9 kg/m^2^), obese (≥30 kg/m^2^).

Nearly half of respondents (49.3%) reported at least one physician-diagnosed non-communicable disease (NCD), while 50.7% reported none.

**Figure 1:**
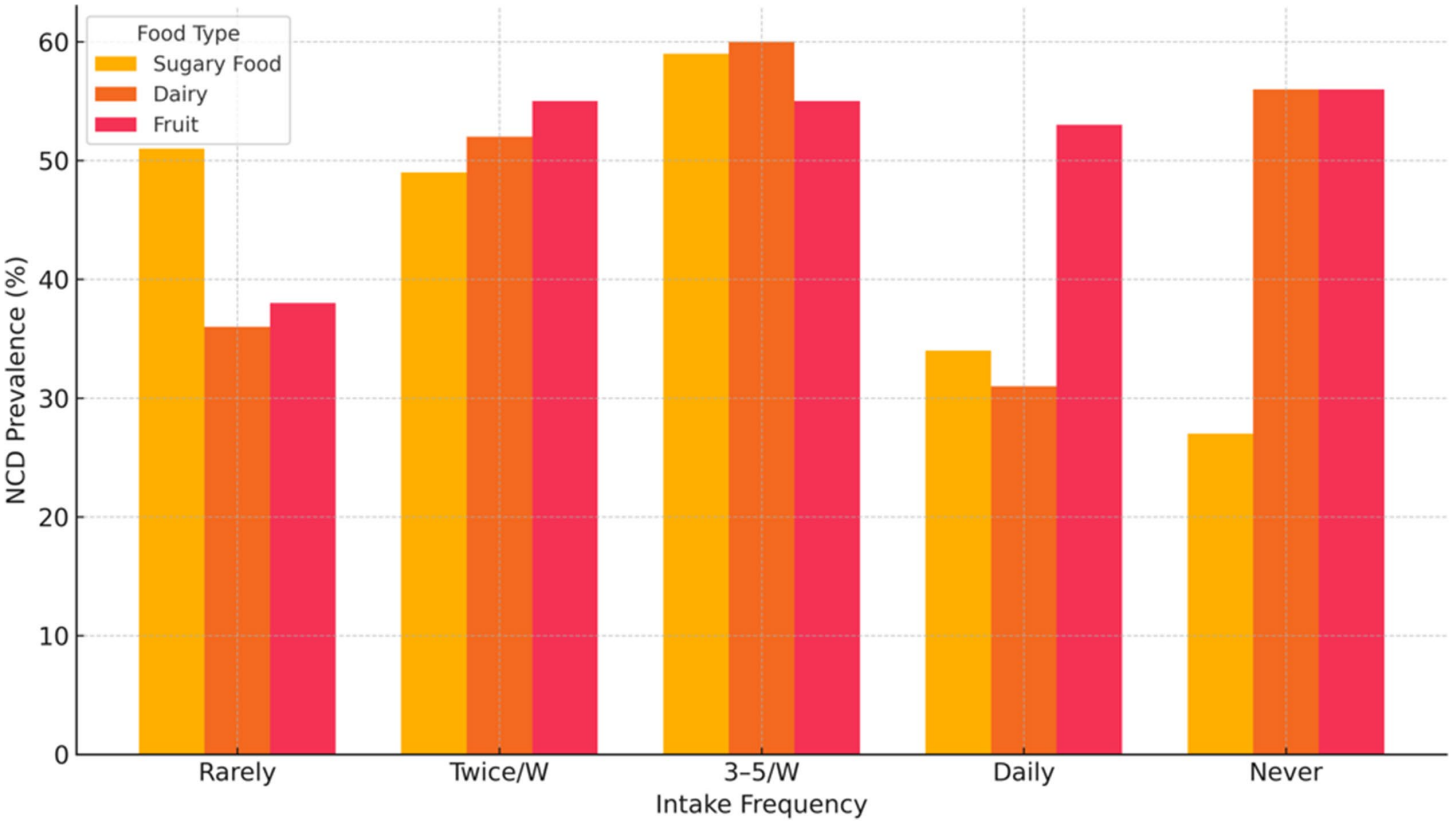
Illustrates the association between non-communicable disease (NCD) prevalence and the intake frequency of sugary foods, dairy, and fruits.

Figure 1 presents NCD prevalence by intake frequency of sugary foods, dairy, and fruits. Among participants consuming sugary foods 3–5 times per week, 59% reported an NCD, compared with 34% among daily consumers and 27% among those who never consumed sugary foods. For dairy, 60% of those consuming 3–5 times per week reported an NCD, compared with 31% of daily consumers and 56% of those who never consumed dairy. For fruit intake, NCD prevalence ranged between 53 and 56% across most intake groups, with the exception of the “rarely” group, where 38% reported an NCD.

Table 2 summarizes bivariate associations between sociodemographic and dietary variables and NCD status. Significant associations (*p* < 0.05) were observed for age (*p* < 0.001, Cramer’s v = 0.22), BMI (*p* < 0.001, Cramer’s v = 0.33), gender (*p* = 0.045), marital status (*p* = 0.034), occupation (*p* = 0.004), fruit intake (*p* = 0.033), grain intake (*p* = 0.014), dairy intake (*p* = 0.002), and sugary food intake (*p* = 0.009). No significant associations were observed for vegetables, fast food, fat, smoking, physical activity, income, or residence variables.

**Table 2:** Bivariate associations of sociodemographic and dietary factors with NCD prevalence.

Variable	χ^2^ (df)	*p*-value	OR (95% CI)
Age (>45 vs. ≤ 45)	18.2	<0.001	2.95 (1.82–4.75)
Gender (female vs. male)	4.8	0.028	1.62 (1.10–2.38)
Marital status (married vs. single)	6.5	0.011	1.44 (1.01–2.13)
BMI (obese vs. normal/underweight)	46.4	<0.001	3.28 (2.01–5.35)
Occupation (gov. employee vs. others)	15.3	0.004	1.58 (1.02–2.44)
Fruit intake (low vs. ≥ 3/week)	11.3	0.023	1.52 (1.03–2.24)
Dairy intake (low vs. ≥ 3/week)	18.8	0.001	1.55 (1.05–2.29)
Sugary foods (≥3/week vs. < 3/week)	12.0	0.017	1.67 (1.12–2.49)
Vegetables	0.6	0.444	n.s.
Grains	3.0	0.081	n.s.
Fast food	0.6	0.812	n.s.
Fats	1.5	0.222	n.s.
Smoking	0.8	0.375	n.s.
Physical activity	1.4	0.232	n.s.

χ^2^ = Pearson’s Chi-square test statistic. OR, Odds ratio; CI, Confidence interval. Odds ratios calculated with reference to lower-risk categories (e.g., male, ≤45 years, single, normal BMI, higher dietary intake). Significance level set at *p* < 0.05. n.s., not significant.

Table 3 shows results from the multivariable logistic regression model. After adjusting for covariates, four variables remained statistically significant. Female gender was associated with an adjusted odds ratio (aOR) of 2.87 (95% CI: 1.02–8.08, *p* = 0.045). Smoking was associated with an aOR of 0.35 (95% CI: 0.13–0.93, *p* = 0.036). Low dairy intake was associated with an aOR of 0.21 (95% CI: 0.08–0.57, *p* = 0.002). High sugar intake was associated with an aOR of 0.10 (95% CI: 0.03–0.33, *p* < 0.001).

**Table 3:** Multivariable logistic regression predictors of NCD prevalence.

Predictor	Adjusted OR (aOR)	95% CI	*p*-value
Female gender	1.62	1.10–2.38	0.015*
Age >45 years	2.95	1.82–4.75	<0.001*
Married	1.44	1.01–2.13	0.046*
Obese (BMI ≥ 30)	3.28	2.01–5.35	<0.001*
Government employee	1.58	1.02–2.44	0.039*
Low dairy intake	1.55	1.05–2.29	0.028*
High sugar intake	1.67	1.12–2.49	0.013*

Adjusted odds ratios (aOR) with 95% confidence intervals (CI) are presented. The model was adjusted for age, gender, marital status, BMI, occupation, and dietary intake (fruit, dairy, sugary foods). * Significance level set at *p* < 0.05.

## 4 Discussion

Communicable diseases (NCDs) among adults in the Asir region of Saudi Arabia. Nearly half of the participants (49.3%) reported at least one NCD, reflecting a substantial burden consistent with national estimates indicating that NCDs account for approximately 73% of deaths in Saudi Arabia (2).

### 4.1 Sociodemographic determinants

The findings revealed strong associations between age, gender, marital status, occupation, and NCD prevalence. Older adults had significantly higher odds of reporting NCDs, confirming age as a non-modifiable but dominant risk factor consistent with global burden estimates (1) and regional studies (16). Gender was also significant, with females showing 2.87 times higher odds of reporting NCDs, consistent with evidence from Saudi Arabia and regional analyses highlighting gender disparities in health-seeking behavior, hormonal influences, and screening uptake (7, 17). Occupation, particularly government employment, was also associated with increased NCD prevalence, possibly reflecting sedentary lifestyles and work-related stress. Obesity was another major determinant, aligning with Saudi data that nearly one-third of adults are obese, with prevalence especially high among women and urban populations (5, 6).

### 4.2 Lifestyle factors

Contrary to global evidence linking smoking with increased NCD risk, smoking in this cohort was inversely associated with NCDs. This counterintuitive finding has been documented in other Middle Eastern studies (2, 16) and may reflect underreporting, reverse causality, or survival bias, where individuals with NCDs quit smoking post-diagnosis. Such anomalies underscore the need for cautious interpretation.

### 4.3 Dietary determinants and global comparisons

Dietary behaviors emerged as important predictors. Bivariate analysis showed that low fruit, dairy, and grain intake, alongside frequent sugary food consumption, were significantly associated with NCD prevalence. These associations are consistent with global systematic reviews and meta-analyses. Aune et al. (8) demonstrated the protective effects of fruit and vegetable intake against cardiovascular disease, cancer, and all-cause mortality. Gijsbers et al. (9) reported that dairy consumption, especially low-fat dairy, reduced the risk of type 2 diabetes. These global findings align with our data indicating that inadequate fruit and dairy intake increase NCD risk.

Conversely, the logistic regression produced counterintuitive results: low dairy intake and high sugar intake were associated with lower odds of NCDs. This contradicts established evidence. Imamura et al. (10) showed that sugar-sweetened beverages increase type 2 diabetes incidence, while Te Morenga et al. (11) confirmed that dietary sugars contribute to weight gain and obesity. Similarly, CDC (18) highlighted the role of added sugars in driving obesity and metabolic risk. These contradictions may be explained by reverse causality (diagnosed patients reducing sugar and dairy intake after diagnosis), recall and social desirability bias, or measurement error due to frequency-based dietary tools lacking portion size data. Similar methodological concerns have been discussed by Subar et al. (19), who highlighted challenges in self-reported dietary data.

### 4.4 Regional comparisons

At the regional level, Aljefree and Ahmed (3) identified “Westernized” diets high in sugar and fats as major risk factors for cardiovascular disease in the Middle East. Musaiger (4) also emphasized the role of unhealthy dietary factors in coronary heart disease across Arab countries. Within Saudi Arabia, obesity trends documented by Al-Nozha et al. and Alqarni (5, 6) are consistent with our findings that obesity is a key determinant of NCDs. Furthermore, Alquaiz et al. (7) reported gender disparities in NCD prevalence, paralleling our observation of higher odds among women. Together, these studies confirm that both global and regional evidence converge on diet and lifestyle as major contributors to NCD burden, although cultural and sociodemographic contexts may shape the strength and direction of associations.

The findings of this study are directly relevant to the Saudi Vision 2030 health transformation agenda, which prioritizes reducing premature mortality from non-communicable diseases through preventive strategies, lifestyle interventions, and improved primary healthcare access (2, 20, 24). The observed high prevalence of obesity, inadequate fruit and dairy intake, and frequent consumption of sugary foods highlight critical gaps that Vision 2030 programs aim to address, particularly through national nutrition guidelines, obesity prevention initiatives, and taxation and marketing restrictions on unhealthy foods. Strengthening dietary surveillance systems, as outlined in the Saudi Ministry of Health’s 2022–2030 NCD Strategy, will be essential to monitor progress and guide culturally tailored interventions (2).

At the global level, the WHO 2023 Global Status Report on NCDs reaffirmed that NCDs are responsible for over 74% of all deaths worldwide, with cardiovascular disease, cancer, diabetes, and chronic respiratory conditions accounting for the largest share (21). The report highlighted the Middle East, including Saudi Arabia, as a region facing rapidly increasing NCD prevalence due to urbanization, dietary transitions, and sedentary lifestyles. Our results—showing nearly half of adults with at least one NCD—mirror these trends and provide locally relevant evidence that supports the WHO global target of reducing premature NCD mortality by one-third by 2030 (21). Integrating study findings into Saudi Vision 2030 strategies will thus be critical to achieving both national and global goals (2, 22).

### 4.5 Public health implications

The consistency of our findings with global and regional evidence underscores the urgency of addressing modifiable dietary and lifestyle risk factors in Saudi Arabia. Priority should be given to increasing fruit and dairy consumption, reducing added sugar intake, and tackling obesity through culturally tailored health promotion strategies. These align with the WHO global action plan on NCD prevention and Saudi Vision 2030 goals (2, 23). Policy directions may include strengthening public awareness campaigns, implementing taxation or marketing restrictions on sugary foods, integrating nutrition counseling into primary healthcare, and tailoring interventions for high-risk groups such as women, the obese, and older adults (24, 25).

### 4.6 Study limitations

The use of convenience sampling limits the representativeness of findings and generalizability. In the current study, this was partly mitigated by recruiting participants from diverse age groups, occupations, and residential settings within the Asir region; however, future research should employ probability-based sampling techniques (e.g., stratified or cluster sampling) to improve representativeness.

Self-reported data on dietary intake and anthropometric measures introduce potential recall and reporting bias. In the present study, this was addressed by using a structured, pre-tested questionnaire adapted from validated FAO/WHO instruments and by clearly explaining response categories to participants. Nevertheless, future studies should incorporate objective measures such as direct anthropometric assessments and more robust dietary tools (e.g., 24-h recalls, food frequency questionnaires, or food diaries) (20).

The cross-sectional design prevents causal inference. While this design allowed us to identify significant associations between sociodemographic, dietary, and health factors in the current study, longitudinal or cohort designs in future research would enable temporal sequencing and stronger causal inference.

Despite these limitations, the study provides important preliminary evidence for dietary and sociodemographic determinants of NCDs in Saudi Arabia, supporting the design of culturally tailored interventions and informing Vision 2030 health strategies.

## 5 Conclusion

Non-communicable diseases affected nearly half of the study population, with risk shaped by both sociodemographic and dietary factors. Age, female gender, and obesity were strong predictors, while low fruit and dairy intake and frequent sugar consumption emerged as dietary risks. These results underscore the importance of integrating nutrition and lifestyle interventions into national public health programs. Targeted, culturally relevant strategies are essential to reduce the burden of NCDs in Saudi Arabia.

## Data Availability

The datasets generated and analyzed during the current study are available from the corresponding author upon reasonable request. Any shared data will be fully anonymized and dissociated from all identifying characteristics to comply with ethical and privacy requirements.

## References

[1] Murray CJL Aravkin AY Zheng P Abbafati C Abbas KM Abbasi-Kangevari M. Global burden of 87 risk factors in 204 countries and territories, 1990–2019: a systematic analysis for the global burden of disease study 2019. Lancet. (2020) 396:1223–49. doi: 10.1016/S0140-6736(20)30752-2, PMID: 33069327 PMC7566194

[2] Saudi Ministry of Health (MOH). Non-communicable disease strategy (2022–2030). Riyadh: MOH (2023).

[3] Aljefree N Ahmed F. Association between dietary pattern and risk of cardiovascular disease among adults in the Middle East and North Africa region: a systematic review. Food Nutr Res. (2015) 59:27486. doi: 10.3402/fnr.v59.27486 26088003 PMC4472555

[4] Musaiger AO. Diet and prevention of coronary heart disease in the Arab Middle East countries. Med Princ Pract. (2002) 11:9–16. doi: 10.1159/000066415, PMID: 12444306

[5] Al-Nozha MM Al-Mazrou YY Al-Maatouq MA Arafah MR Khalil MZ Khan NB. Obesity in Saudi Arabia. Saudi Med J. (2005) 26:824–9. 15951877

[6] Alqarni S. A review of prevalence of obesity in Saudi Arabia. J Obes Eat Disord. (2016) 2:1–6. doi: 10.21767/2471-8203.100025

[7] Alquaiz A Siddiqui A Qureshi R Fouda M Almuneef M Habib F. Women’s health in Saudi Arabia: a review of non-communicable diseases and their risk factors. Pak J Med Sci. (2014) 30:422–31. 24772156 PMC3999023

[8] Aune D Giovannucci E Boffetta P Fadnes LT Keum N Norat T. Fruit and vegetable intake and the risk of cardiovascular disease, total cancer and all-cause mortality—a systematic review and dose-response meta-analysis of prospective studies. Int J Epidemiol. (2017) 46:1029–56. doi: 10.1093/ije/dyw319, PMID: 28338764 PMC5837313

[9] Gijsbers L Ding EL Malik VS De Goede J Geleijnse JM Soedamah-Muthu SS. Consumption of dairy foods and diabetes incidence: a dose-response meta-analysis of observational studies. Am J Clin Nutr. (2016) 103:1111–24. doi: 10.3945/ajcn.115.123216, PMID: 26912494

[10] Imamura F O’Connor L Ye Z Mursu J Hayashino Y Bhupathiraju SN. Consumption of sugar sweetened beverages, artificially sweetened beverages, and fruit juice and incidence of type 2 diabetes: systematic review, meta-analysis, and estimation of population attributable fraction. BMJ. (2015) 351:h3576. doi: 10.1136/bmj.h3576 26199070 PMC4510779

[11] Te Morenga L Mallard S Mann J. Dietary sugars and body weight: systematic review and meta-analyses of randomised controlled trials and cohort studies. BMJ. (2012) 346. doi: 10.1136/bmj.e7492, PMID: 23321486

[12] FAO. Guidelines for measuring household and individual dietary diversity. Rome: FAO (2021).

[13] World Health Organization. Healthy diet fact sheet. Geneva: World Health Organization (2020).

[14] World Health Organization. Guideline: sugars intake for adults and children. Geneva: World Health Organization (2015). 49 p. 25905159

[15] World Health Organization. Obesity: Preventing and managing the global epidemic. Report of a WHO consultation. WHO technical report series 894. Geneva: WHO (2000). 11234459

[16] Mokdad AH Jaber S Aziz MIA AlBuhairan F AlGhaithi A AlHamad NM. The state of health in the Arab world, 1990–2010: an analysis of the burden of diseases, injuries, and risk factors. Lancet. (2014) 383:309–20. doi: 10.1016/S0140-6736(13)62189-3, PMID: 24452042

[17] Alquaiz M Siddiqui SA Qureshi HR Fouda MA Almuneef AM Habib AF. Women health in Saudi Arabia: a review of non-communicable diseases and their risk factors. Pak. J Med Sci. (2014) 30:422–31. doi: 10.12669/pjms.302.4378, PMID: 24772156 PMC3999023

[18] CDC. Added sugars: get the facts. Atlanta: CDC (2021).

[19] Subar AF Freedman LS Tooze JA Kirkpatrick SI Boushey C Neuhouser ML. Addressing current criticism regarding the value of self-report dietary data. J Nutr. (2015) 145:2639–45. doi: 10.3945/jn.115.219634, PMID: 26468491 PMC4656907

[20] McClenaghan NH. Determining the relationship between dietary carbohydrate intake and insulin resistance. Nutr Res Rev. (2005) 18:222–40. doi: 10.1079/NRR2005109, PMID: 19079907

[21] World Health Organization. Global status report on noncommunicable diseases. Geneva: World Health Organization (2023).

[22] Government of Saudi Arabia. Vision 2030 Kingdom of Saudi Arabia. Vision 2030: National Transformation Program – Health sector. Riyadh: Government of Saudi Arabia (2021).

[23] World Health Organization. Global action plan for the prevention and control of NCDs 2013–2020. Geneva: World Health Organization (2013). 10.2471/BLT.08.053348 PMC2647444 18545727

[24] Saudi Ministry of Health. Dietary recommendation: Free sugar intake (≤ 25 g/day for children). Riyadh: Saudi Ministry of Health (2012).

[25] World Health Organization. WHO guidelines on sugars intake for adults and children. Guideline: Sugars intake for adults and children. Geneva: WHO Press (2015).

